# An Adaptive Knee Brace Is Associated With Improved Dynamic Stabilization Compared With a Standard Passive Brace in Anterior Cruciate Ligament‐Deficient Men During a Functional Hop Task

**DOI:** 10.1002/ars2.70133

**Published:** 2026-11-08

**Authors:** Shai Greenberg, Roni Gottlieb

**Affiliations:** ^1^ School of Human Movement and Sport Sciences The Levinsky‐Wingate Academic College (Wingate Campus) Netanya Israel

## Abstract

**Purpose:**

To compare the dynamic knee stabilization effects of an adaptive dynamic brace (ADB) with those of a standard passive brace in anterior cruciate ligament (ACL)‐deficient men.

**Methods:**

ACL‐deficient participants were included. The subjects performed 3 single‐leg functional hop tests: drop jump, crossover hop, and 90° rotational hop. Each task was performed once while wearing the ADB and once while wearing a traditional passive brace. Three‐dimensional kinematic data were collected using an 8‐camera optoelectronic motion‐capture system. Repeated‐measures analysis of variance was used to evaluate the effects of brace condition and time. An a priori power analysis indicated that a minimum of 12 participants were required.

**Results:**

Twelve ACL‐deficient participants were included (age: 30.2 ± 3.4 years; time since injury: 32 ± 6 months; ACL‐Return to Sport after Injury score: 70 ± 3). No significant differences in knee flexion angles were observed between braces. Knee flexion during landing was significantly lower in the crossover hop compared with the drop jump and rotational hop tasks (*P* < .01). During the drop jump task, the ADB significantly reduced knee valgus angles (*P* < .01). In the crossover hop task, the ADB significantly reduced both knee valgus and rotational angles (*P* < .01). During the rotational hop task, a significant brace‐by‐time interaction was observed for knee valgus (*P* < .05) and rotational angles (*P* < .05).

**Conclusions:**

The ADB showed improved dynamic stabilization particularly during high‐risk landing and rotational movement compared with a standard passive brace.

**Clinical Relevance:**

The ADB may enhance the effectiveness of rehabilitation strategies in ACL‐deficient individuals by improving dynamic knee stability during functional activities.

## Graphical abstract

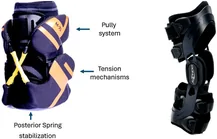

Anterior cruciate ligament (ACL) tears are among the most common and severe injuries in sports, with more than 250,000 cases reported annually in the United States.^1^, ^2^ Between 2004 and 2014, a 22% increase in ACL injuries was observed among young athletes aged 13 to 17 years.^3^ In most cases, reconstruction surgery is required, followed by approximately 9 months of rehabilitation.^4^ The rising incidence of ACL injuries,^3^ their occurrence at increasingly younger ages, the high risk of reinjury,^5^, ^6^ and persistent postrecovery performance deficits have generated growing interest in injury‐prevention strategies.^7^

One commonly employed preventive measure is bracing. Surveys show that 55% to 65% of orthopaedic physicians in the National Football League and National Collegiate Athletic Association recommend braces after isolated ACL tears.^1^, ^8^ However, the biomechanical and clinical effectiveness of bracing remains controversial. Some studies conducted in patients following ACL reconstruction suggest that bracing can restrict knee valgus during landing,^9^ reduce knee‐joint impact,^10^ enhance proprioception,^11^ and limit knee rotation.^12^, ^13^ Conversely, other studies have reported that bracing has no significant effect than soft brace on knee valgus,^14^ has no effect on knee rotation,^15^ and may even increase knee rotation among ACL‐deficient subject.^16^ Prospective studies did not show any prevention effect between ACL brace and sleeves.^17^, ^18^

Several factors may explain the limited protective effect of conventional bracing. ACL injury mechanisms are complex and variable involving multiplanar forces such as anterior tibial translation, valgus collapse, and internal/or external rotation. The most common injury mechanisms: combines tibia rotates externally while the hip rotates internally.^19^, ^20^ In addition, ACL loading is maximal when the knee is flexed at approximately 30°, and injuries usually occur during high‐velocity explosive movements such as landing or cutting maneuvers. Recently, more advanced dynamic braces have been introduced,^21^, ^22^ which have shown reductions in meniscal strain and ACL loading during daily activities. However, these effects have not been consistently observed under functional athletic conditions.

To address these limitations, we propose an adaptive dynamic brace (ADB). This brace uses knee flexion motion between 15° and 45° during landing or directional changes to generate a posteriorly directed force on the tibia while simultaneously restricting rotation. In a previous study performed on healthy subjects using pressure sensors, the ADB produced significantly greater stabilization forces on the tibia during orthopaedic stability tests and functional and explosive tasks compared with a static brace.^23^

The purpose of this study was to compare the dynamic knee stabilization effects of an ADB with those of a standard passive brace in ACL‐deficient individuals. We hypothesized that, compared with a standard passive brace, the ADB would significantly reduce knee rotational motion during landing and cutting maneuvers in ACL‐deficient individuals, particularly within lower knee flexion angles, thereby providing greater functional dynamic stabilization.

## METHODS

### Participants

This study was approved by Tel Aviv University (register number: TLV 0163‐23). Written informed consent was obtained from all participants prior to enrollment. All participants underwent a standardized clinical evaluation by a sports orthopaedic surgeon and completed the ACL‐Return to Sport after Injury questionnaire before enrollment.^24^ Participants were recruited between March 2024 and March 2025.

Inclusion criteria were as follows: at least 6 months postinjury without surgical reconstruction; completion of a minimum of 3 months of supervised exercise rehabilitation program; performing regular physical activity but because of instability episodes failed to return to preinjury sports activity, particularly including cutting or directional change maneuvers; and an ACL‐Return to Sport after Injury questionnaire inclusion criterion score between 50 and 80 out of 100. Age 18 to 40 years, MRI‐confirmed complete ACL tear sustained through a noncontact mechanism positive Lachman test (≥grade 1) compared with the contralateral knee, preserved posterior, collateral, and posterolateral stability, full knee range of motion. Exclusion criteria were as follows: contralateral knee injury, meniscal defects, other musculoskeletal or neurological impairments of the lower limbs that could compromise stability (e.g., ankle instability, prior ankle surgery, hip pathology, or neurological deficits affecting strength), and intra‐articular fractures of the lower extremities (e.g., tibial plateau or ankle joint fractures).

### 3‐Dimensional Motion Analysis Protocols

#### General Procedures

Lower‐limb kinematics were recorded using an 8‐camera motion capture system (Vicon, Oxford Metrics) operating at 200 Hz. A full‐body Plug‐in Gait marker set was used, with reflective markers placed bilaterally on key anatomical landmarks: anterior/posterior superior iliac spines, greater trochanters, femoral epicondyles, tibial tuberosities, malleoli, calcanei, and first and fifth metatarsal heads. Additional marker clusters were secured to the thighs and shanks to minimize soft tissue artifacts.

Marker trajectories were filtered using a fourth‐order zero‐lag Butterworth low‐pass filter at 6 Hz.^25^ Initial contact was defined as the first frame in which the foot visibly contacted the ground. Kinematic variables extracted from initial contact to peak knee flexion included the knee flexion angle, knee valgus/varus angle, and tibial internal/external rotation.

Testing was supervised by a physiotherapist with >15 years of clinical experience. Braces were fitted according to the manufacturer's guidelines and adjusted for comfort and stability. Participant practiced task until confidence. Each task was repeated 3 times. For safety, the order of tasks was arranged by increasing the physical load, and progression was permitted only after successful completion of the prior task.

### Functional Hop Tests

#### Drop Jump (DJ)—Vertical Loading

Participants stood on a 30‐cm box and, on command, stepped forward to land on 1 leg, immediately performed a maximal vertical jump, and landed again on the same leg. Three successful trials were required, defined as stable landings without hesitation or additional steps.^26^

#### Crossover Single‐Leg Hop Horizontal/Rotational Loading

Participants began in a single‐leg stance 50 to 70 cm lateral to a centerline. The exact starting position was determined individually based on pretrial attempts to maximize the participant's performance. Participants hopped forward crossing the line to land on the same leg and then hopped back across to the other side. They were instructed to stabilize for at least 2 seconds after landing. A successful trial required 2 clean line crossings and stable landings.^27^

#### Ninety‐Degree Rotational Hop (RH)—Vertical and Rotational Loading

Participants stood 40 to 60 cm from a measurement tape. The exact starting position was determined individually based on pretrial attempts to maximize the participant's performance, and participants hopped forward on 1 leg while simultaneously rotating 90° in the air, landing facing perpendicular to the starting orientation. Immediately, they performed a vertical jump and landed again on the same leg, stabilizing for 2 seconds. Successful trials required full rotation and stable landings.^28^

### ACL Braces

Each participant completed the 3 hop tests twice in random order: once wearing a commercial static ACL brace (DonJoy ACL Everyday Knee Brace, DJO, interface length: 32 cm) and once wearing a prototype variable adaptive brace (OnCourt R, Israel, interface length: 22 cm) (Figure 1).

**FIGURE 1:**
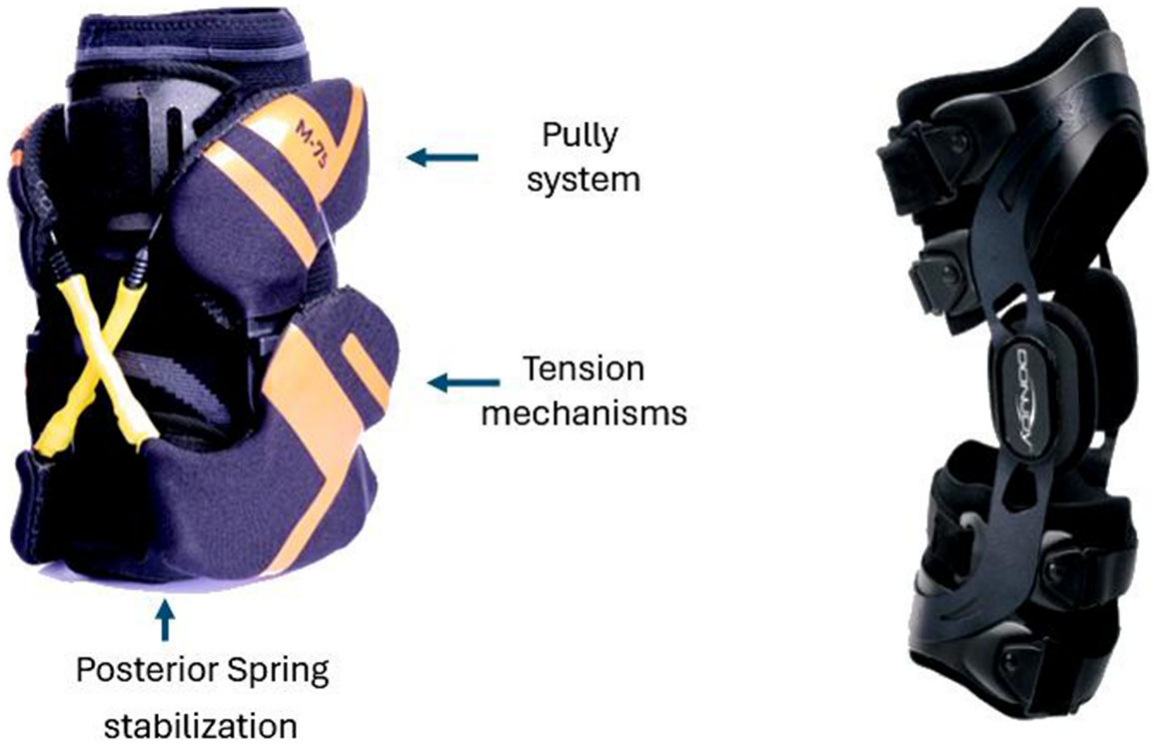
Brace used in the experiment. Left: adaptive dynamic brace. Right: static ACL brace. (ACL, anterior cruciate ligament.)

### Adaptive Dynamic Brace

Structure: shortened plastic frame with a femoral strap and tibial strap. At the hinge, an eccentric tension mechanism connected to 2 posteriorly crossed cables attached distally to calibrated springs (Nordia Springs, Israel). Each 1‐cm spring elongation generated ~20 N of force, based on prior in vitro validation.^29^ Action: during knee flexion beginning at 15°, hinge motion increased cable traction, stretching the springs and generating a posteriorly directed stabilizing force with a counter‐rotational component. Force peaked at ~30° flexion and decreased gradually, approaching 0 by 50°.

### Statistical Analysis

Data were analyzed using SPSS (IBM, Armonk, NY), version 26. The following kinematic parameters were calculated and compared for each participant and test: knee flexion angle, valgus/varus angle, and internal/external rotation. Data were evaluated throughout the landing phase, from initial contact (the moment the participant's foot touched the ground) to maximal knee flexion. An average of the 3 successful attempts was calculated. A 1‐way repeated‐measures analysis of variance was used to assess brace condition effects, and a 2‐way repeated‐measures analysis of variance was performed to examine time effects. Because all participants completed all experimental conditions, no missing data were present. Data normality was assessed using the Shapiro‐Wilk test. Statistical significance was set at *P* < .05. Effect estimates are reported with their corresponding 95% confidence intervals, where appropriate. An a priori power analysis was conducted using G*Power software for a repeated‐measures analysis of variance (within factors). The analysis assumed a medium effect size (Cohen's *f* = 0.25), a significance level of *α* = 0.05, and a desired statistical power of 1 − *β* = 0.80. The calculation indicated that a minimum sample size of 12 participants was required.

## RESULTS

### Subject Characteristics

Twelve male participants with ACL deficiency were included, involving 7 right knees and 5 left knees (mean ±standard deviation: age 30.2 ± 3.4 years; time since injury 32 ± 6 months; height 1.76 ± 0.2 m; body mass 78 ± 3 kg; ACL‐Return to Sport after Injury score 70 ± 3).

### Safety

All participants successfully completed the test procedures without experiencing symptoms of knee instability, pain, or discomfort while using either brace.

### Drop Jump

No statistical differences were observed between the 2 braces in knee flexion during landing during initial contact (Table 1). Wearing the ADB resulted in a significant reduction in knee valgus from initial contact and throughout landing compared with the static brace (*P* < .01; Figure 2). Knee rotational angles at initial contact were also lower with the ADB compared with the static brace, but this difference did not reach statistical significance. As knee flexion increased, this effect was diminished and rotational angles were similar.

**TABLE 1:** Kinematic Knee Angle Average at Initial Contact

**Test**	**Sagittal**		**Frontal**		**Transverse**	
	**S**	**D**	**S**	**D**	**S**	**D**
DJ	35.4 ± 1.3	33.4 ± 1.2	6.4 ± 1.2	4.9 ± 2.2*	7.2 ± 1.5	6.9 ± 1.4
CO	31.5 ± 0.9**	30.2 ± 0.8*	7.4 ± 1.7	5.8 ± 2.2*	5.8 ± 4.1	1.6 ± 2.1*
RH	36.1 ± 1.5	36.3 ± 1.7	5.1 ± 1.4	4.3 ± 1.5	6.4 ± 1.6	5.2 ± 1.4

*Note*: Frontal plane: positive values indicate valgus alignment. Transverse plane: positive values indicate internal rotation. Data are presented as mean ± SD.

CO, crossover hop; D, adaptive dynamic brace; DJ, drop jump; RH, rotational hop; S, static brace; SD, standard deviation.

∗ *P* < .01 significance between braces.

∗∗ *P* < .01 significance between hops.

**FIGURE 2:**
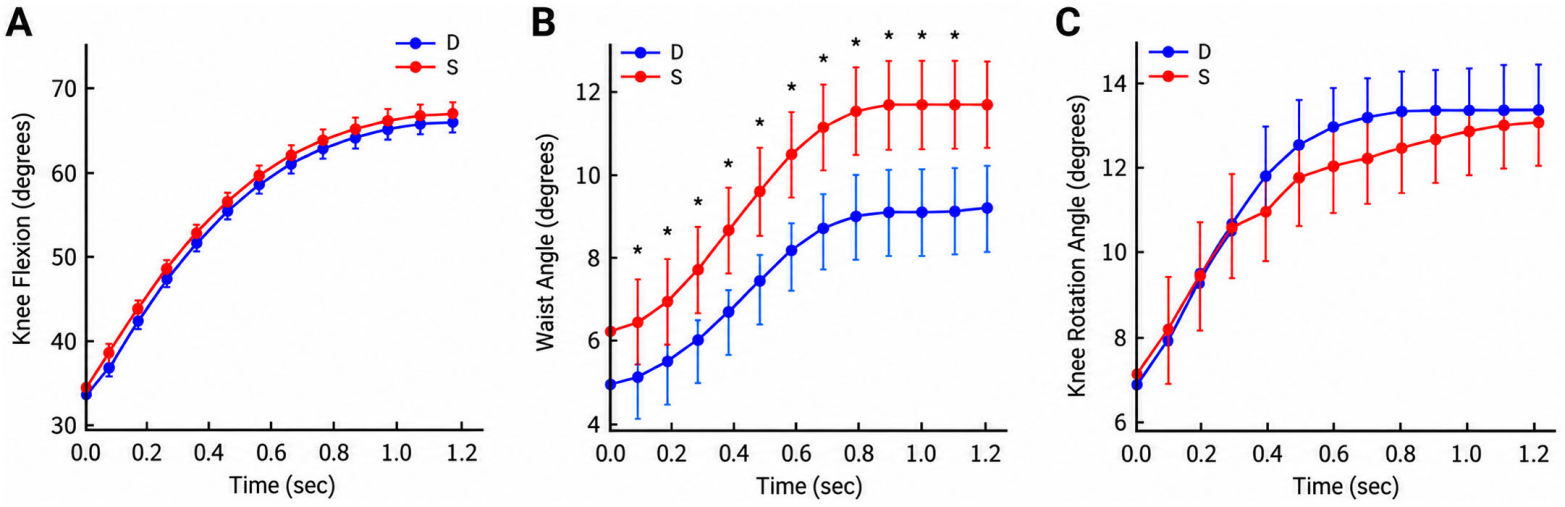
Kinematic parameters during the DJ: (A) sagittal plane, (B) valgus plane, and (C) rotational plane. Blue: ADB; red: static brace. ^*^ *P* < .05 difference between braces. (ADB, adaptive dynamic brace; DJ, drop jump.)

### Crossover Hop

No significant differences were observed between the braces in the knee flexion angle (*P* = .80). However, knee flexion angles were significantly lower in both braces compared with the DJ and RH tests, at initial contact (Table 1) and during all landing (*P* < .01) (Figure 3). Wearing the ADB resulted in significantly lower knee valgus and rotational angles from initial contact throughout landing compared with the static brace (*P* < .01; Figure 4).

**FIGURE 3:**
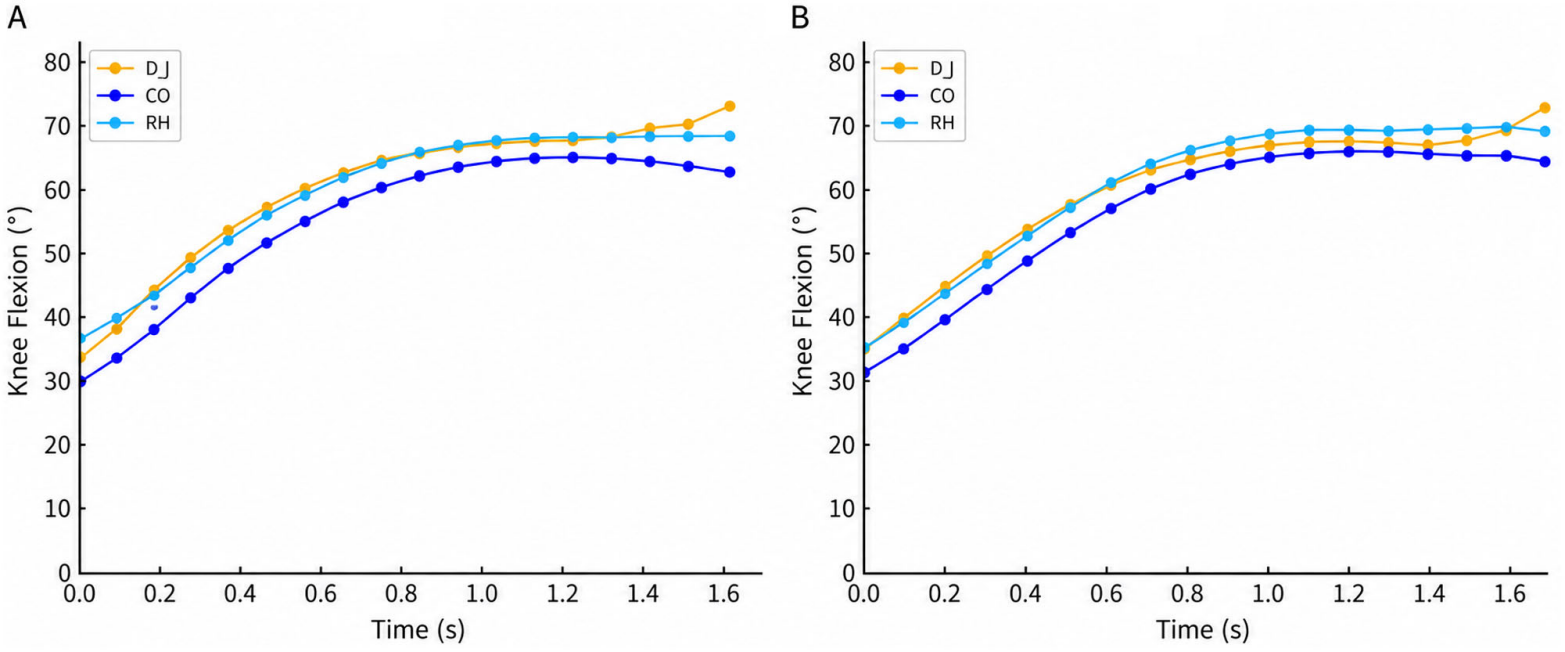
Knee flexion angles during landing at 3 tests. (A) Static brace. (B) Adaptive dynamic brace. (CO, crossover hop; DJ, drop jump; RH, rotational hop.)

**FIGURE 4:**
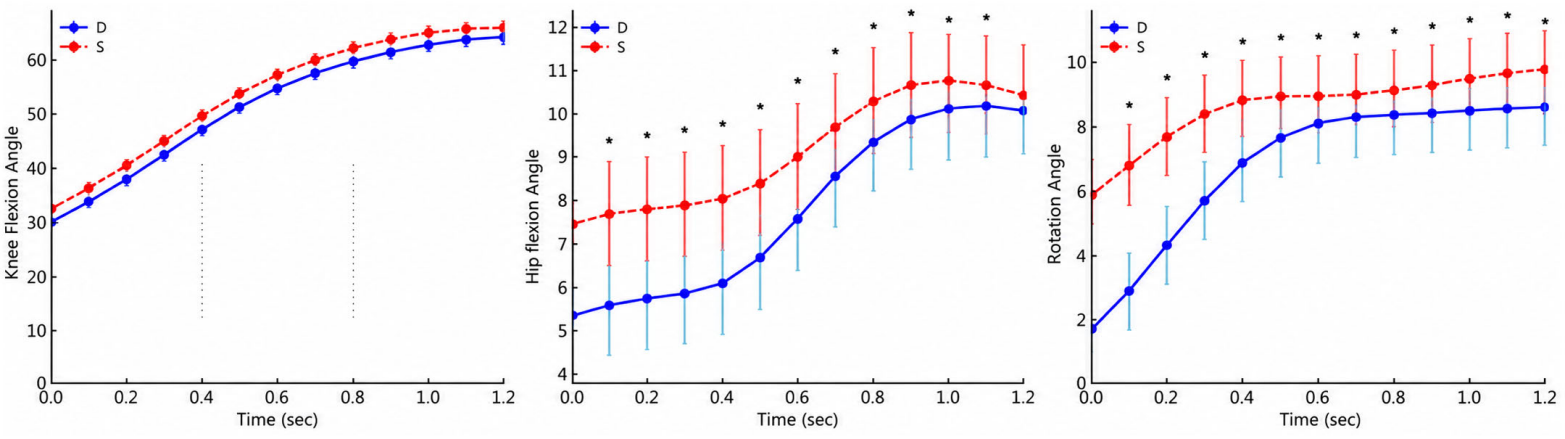
Kinematic parameters during the CO: Left: sagittal plane, middle: valgus plane, and right: rotational plane. Blue: ADB; red: static brace. ^*^ *P* < .01 difference between braces. (ADB, adaptive dynamic brace; CO, crossover hop.)

### Ninety‐Degree RH

No significant differences were observed between the braces in the knee flexion angle during landing. Wearing the ADB was associated with reduced, although nonsignificant, knee valgus and rotational angles at initial contact (Table 1) and during low flexion angles, up to 40° of knee flexion (Figure 5). These effects progressively diminished as knee flexion increased toward 50°. Above 65° of knee flexion, the static brace showed lower valgus and rotational angles compared with the ADB. These changes throughout landing resulted in only significant brace‐by‐time interactions for both knee valgus and rotational angles (*P* < .05).

**FIGURE 5:**
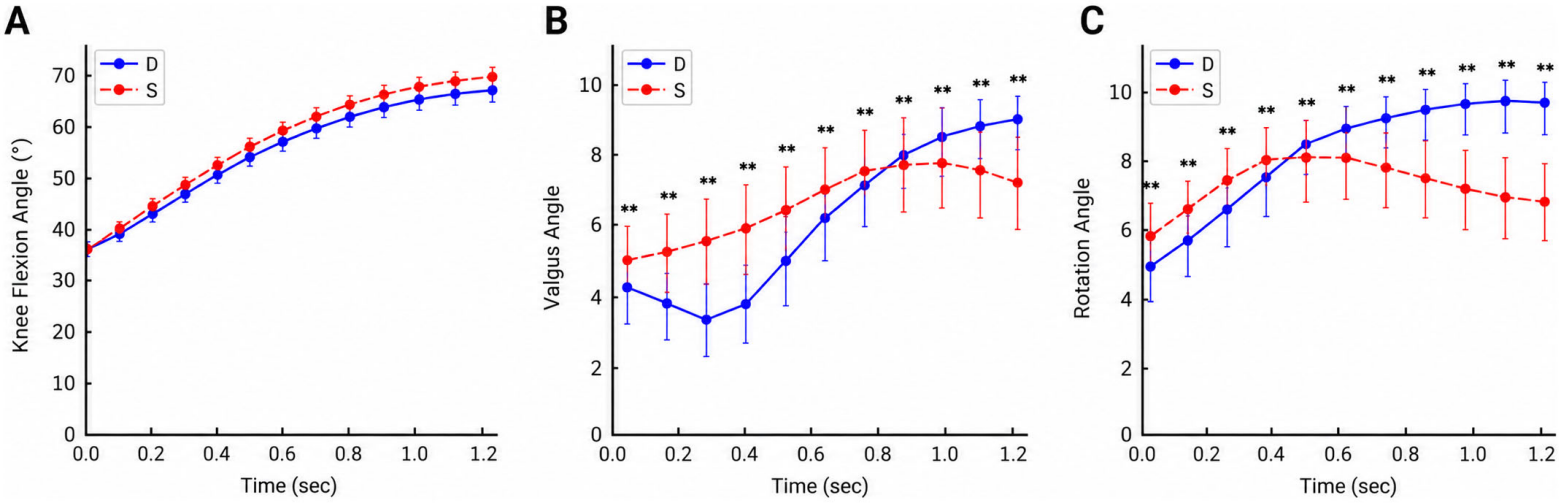
Kinematic parameters during the RH: (A) sagittal plane, (B) valgus plane, and (C) rotational plane. Blue: ADB; red: static brace. ^*^^*^ *P* < .05 difference between braces and time interaction. (ADB, adaptive dynamic brace; RH, rotational hop.)

## DISCUSSION

The main findings show that during the crossover hop, a task that reproduces ACL injury mechanisms by combining low knee flexion, valgus, and rotation,^30^, ^31^ wearing the ADB produced a significant protective effect: reduced knee valgus and rotational angles throughout all landing phases. We also found that during DJ landings, the ADB resulted in lower valgus angles compared with the static brace. Furthermore, during hop tests^27^ that induced deeper flexion and rotation, significant differences in the stabilization effects of the 2 braces were observed over time. Specifically, the ADB reduced valgus and rotational angles at initial flexion angles, whereas the static brace showed greater reductions later in the landing phase.

This study compared the effects of an ADB designed to deliver stabilization forces at initial flexion angles associated with a high risk of ACL injury with those of a static commercial brace. Our results align with previous studies,^32^, ^33^, ^34^, ^35^ suggesting that although current static braces may produce measurable kinematic effects during daily activities and early rehabilitation, their protective effects during athletic maneuvers remain less clear. Under high‐demand tasks such as DJs or directional changes, braces have been reported to improve subjective perceptions of stability,^36^ increase knee flexion during landing, and enhance proprioceptive feedback.^35^ However, their direct biomechanical effects on injury‐related stabilization have been inconsistent, largely because of differences in testing protocols. For example, during tasks involving vertical loading at deep flexion angles, or combined vertical and rotational loading in deep flexion,^11^, ^12^ static braces showed significant rotational stabilization effects compared with no brace, but not when compared with soft braces.^13^, ^14^ In contrast, during tasks involving rotational loading at low flexion angles, static braces were not found to produce a stabilizing effect.^15^, ^16^ This is probably why prospective studies performed among athletes^17^, ^18^ found that continuous bracing use reduces overall traumatic knee injuries but not ACL injury rates.

In addition, we found a significant difference between the brace stabilization effect and time interaction. During RH at deep flexion angles (~50°), the static brace provided partial stabilization of both valgus and rotational angles. However, at lower flexion angles, from initial contact (32°) to around ~50°, the ADB showed superior stabilization, reducing both valgus and rotational angles compared with the static brace.

An additional finding supporting the effect of the ADB was the reduction in the valgus angle during DJ landings. This result is also consistent with previous studies reporting^9^ that static braces reduce valgus angles compared with no brace, but these effects were not significant in other studies when the static brace was compared with soft braces.^14^ This implies that the effects of static braces may not result from mechanical restriction, but rather from modifications in muscle activation.^37^ By contrast, the ADB showed a significant effect despite its shorter frame and consequently shorter lever arm compared with the static brace. These findings are consistent with previous investigations,^23^ indicating that the observed motion limitation is attributable to direct dynamic mechanical stabilization rather than neuromuscular modulation. However, electromyography measurements were not performed, making this explanation speculative. The lack of effect of the ADB on rotational angles is because of the moderate rotational demand in this test compared with other test.^30^

The clinical relevance of these findings is highlighted by the characteristics of the study population. Individuals with ACL deficiency typically exhibit greater dynamic knee valgus and rotational instability than healthy controls.^38^ Therefore, the additional external stabilization provided by the ADB may improve dynamic knee stability, which may enhance functional performance and facilitate a safer return to activity.

### Limitations

This study has several limitations. First, only male participants were included, which limits the generalizability of the findings. Second, healthy knees or reconstructed knees were not included as the control group. This decision was based on prior several studies^12^, ^13^, ^15^, ^16^ that failed to show consistent or significant biomechanical effects of passive bracing, leading us instead to use a static commercial brace as the control condition. Third, because of the small sample size and the absence of statistically significant difference for some outcomes, the study may have been underpowered, increasing the risk of type error. Fourth, force plate landing data were not synchronized with the kinematic data. However, because the present study specifically focused on the initial contact phase rather than the entire landing phase, this limitation was considered to have a minimal impact on the primary outcome.

## CONCLUSIONS

The ADB showed improved dynamic stabilization compared with a standard passive brace, particularly during high‐risk landing and rotational movements, by reducing knee valgus and rotational angles.

## DISCLOSURES

The author (S.G.) declares the following financial interests/personal relationships which may be considered as potential competing interests: S.G. filed a patent application related to the ADB evaluated in the study. The other author (R.G.) declares that he has no known competing financial interests or personal relationships that could have appeared to influence the work reported in this article.

## FUNDING

This work was supported by the Israel Innovation Authority (84323).
